# Age-Related Deficits in Memory Encoding and Retrieval in Word List Free Recall

**DOI:** 10.3390/brainsci8120211

**Published:** 2018-11-30

**Authors:** Dorina Cadar, Marius Usher, Eddy J. Davelaar

**Affiliations:** 1 Department of Behavioural Science and Health, University College London, London WC1E 7HB, UK; d.cadar@ucl.ac.uk; 2 Department of Psychology, Tel-Aviv University, Tel-Aviv 69978, Israel; marius@tauex.tau.ac.il; 3 Department of Psychological Sciences, Birkbeck, University of London, London WC1E 7HX, UK

**Keywords:** ageing, memory encoding, memory retrieval, free recall, semantic clustering

## Abstract

Although ageing is known to affect memory, the precise nature of its effect on retrieval and encoding processes is not well understood. Here, we examine the effect of ageing on the free recall of word lists, in which the semantic structure of word sequences was manipulated from unrelated words to pairs of associated words with various separations (between pair members) within the sequence. We find that ageing is associated with reduced total recall, especially for sequences with associated words. Furthermore, we find that the degree of semantic clustering (controlled for chance clustering) shows an age effect and that it interacts with the distance between the words within a pair. The results are consistent with the view that age effects in memory are mediated both by retrieval and by encoding processes associated with frontal control and working memory.

## 1. Introduction

Episodic memory is known to decline with age, and this decline affects some tasks and processes more than others. For example, the largest age-related declines are found in tasks, such as free recall, which depend on retrieval strategies, while smaller deficits are found in recognition memory [1]. This conclusion is supported by studies of free recall [2], which reported that older adults have lower temporal contiguity effects (a reduction in the conditional probability of sequentially reporting items in proximal list positions). This is an effect which is associated with the use of retrieved context to guide subsequent retrieval [3]. Furthermore, an ageing retrieval deficit is suggested by verbal fluency studies, showing that older adults retrieve fewer words in a semantic fluency task, in which as many animal names as possible have to be generated within a fixed time period [4]. As no encoding is required in verbal fluency, this strongly supports an ageing effect on memory retrieval. While such studies suggest that age-related decline in episodic memory is due to a retrieval deficit, encoding deficits have also been demonstrated, as older participants are less likely to form rich, elaborative memory traces [5]. Furthermore, smaller age differences in memory are found when the initial encoding is equated [6].

The ageing deficit has been explained in terms of a reduction in frontal lobe functioning [7]. This hypothesis is supported by significant correlations between neuropsychological measures of frontal lobe functions and memory tests sensitive to ageing [8]. Morphological [9] and neuroimaging studies [10,11] give further support to this hypothesis by showing reduced activation in the left prefrontal cortex (PFC, associated with memory encoding and the processing of semantic information) during memory encoding and semantic processing. Further support for the “ageing as PFC reduction” perspective comes from the observation that like older adults, frontal patients show clear deficits in tests of free recall but not in memory recognition [12,13] and source memory (more than in item memory [14]).

Moscovitch [15] argued that both encoding and retrieval of consciously apprehended information are supported by the medial temporal lobes and hippocampus [16,17,18], and that these processes are under voluntary control. He noted that the frontal lobes operate on these structures and guide the encoding, retrieval, monitoring, and organisation of information: “By operating on the medial temporal and diencephalic system, the frontal lobes act as working-with-memory structures that control the more reflexive medial temporal and diencephalic system and confer a measure of intelligence and direction to it” ([15], p. 8, see also [19]).

Although evidence suggests that ageing affects both encoding and retrieval, a recurrent problem of interpretation is that different tasks and methods are used to provide support in favour of the encoding or retrieval interpretation. Here, we focus on the task of free recall (a single task from which metrics of encoding and retrieval can be extracted) in which the effect of age can be investigated.

Free recall of word list sequences can be used to quantify the contributions of encoding and retrieval processes in ageing. In a typical experimental paradigm, participants memorise a sequence of words consisting of exemplars from a small number of categories (e.g., four words from each of four categories). The words may be presented sequentially in a blocked-by-category fashion or randomized, and the recall is scored both in terms of the total number of correctly reported words and in terms of the degree of semantic clustering (see Section 2.4). One rationale for examining clustering performance is that it could provide a measure of retrieval strategies and memory organisation [20], uncontaminated by differences in total recall. Ageing studies have shown that semantic clustering scores are lowered for older adults and frontal patients compared with controls [20,21], but the evidence on the ageing effect remains mixed [22,23]. However, differences in semantic clustering scores in free recall may reflect both encoding and retrieval processes [24]. For example, memorising a word is easier when a semantically related word was just committed to memory. In that case, the semantics will help to encode the words in an episodic chunk, which during retrieval leads to a clustered output. In the absence of a semantically supported chunk, having just retrieved a word might prime the retrieval of a semantically related word. This would also produce a clustered output. Both processes are sensitive to ageing effects, but relative sensitivities are unclear.

The aim of our study is to estimate whether age deficits in a single task of word free recall are mediated by retrieval, by encoding processes, or by both. Based on previous studies [21,25], we expect to find age-related differences in both total recall and clustering. These studies, however, were able to assess only encoding or retrieval effects, but not both. To dissociate or isolate encoding and retrieval deficits within a single task, we employed a method that is sensitive to associative processes at encoding, while keeping the retrieval demands constant. This design is created by systematically varying the separation between semantic associates within a word list as part of a free recall paradigm, which is adapted from Glanzer [26] (see also [27]), who presented participants with lists made of pairs of weakly associated words (e.g., “stomach–liver”).

The critical manipulation is the separation between associated pair members in the list. There are four conditions: Three related and one unrelated. The related ones are divided into three separation levels, i.e., the number of unrelated intervening words between a pair of associates. Thus, for the related conditions, each list contains one separation condition: separation-0 corresponds to a1, a2, b1, b2...; separation-1 to a1, b1, a2, b2…; and separation-5 to a1, b1, c1, d1, e1, f1, a2, b2…. Using such a design, Glanzer [26] reported that memory recall was higher for lists with associated, compared with unrelated words, and was higher when the associates were separated by fewer unrelated words (small separation). As proposed by Glanzer, and demonstrated in simulation models [27,28], this separation effect can be explained as a result of semantic-associative processes during encoding in a capacity-limited working memory [27,29]. We have shown how encoding processes supported by the prefrontal cortex (PFC) operate on sequences that include an associative structure [24]. Specifically, when members of a pair reside in working memory simultaneously, the associative link between the words is increased (see also [30]), by recruiting category units that are linked with the list context and support subsequent retrieval. Thus, total recall, as well as semantic clustering, are predicted to be higher for word pairs whose members are separated by fewer unrelated words, as they are more likely to co-occupy working memory. Such a differential effect observed with a separation design is thus an encoding effect.

Using this paradigm, the separation-1 condition (i.e., one intervening item between pair members) is critical in demonstrating the age deficits that are in part due to declines in working memory assisted encoding. At separation-1, older participants may be more strongly affected in their recall of related words, because those words were less likely to have been maintained within their reduced working memory. Therefore, if ageing affects memory encoding, we predict an age-related reduction in semantic clustering at separation-1, which will differ from separation-5, as recall of word pairs separated by five intervening words is not assumed to be mediated by working memory. Thus, with this paradigm, we can demonstrate the additional influence of encoding processes on top of retrieval within a single task, based on the separation between associates.

To summarise, we expect age differences at encoding to result in an interaction between age and separation, either on total recall or on clustering, while age differences at retrieval to result in an interaction between age and relatedness with a long-separation (separation-5).

## 2. Materials and Methods

### 2.1. Participants

Forty native English speakers, who reported being in good health, took part in this study. The younger (*n* = 20, age range 19–35, mean 27) and older (*n* = 20, age range 55–65, mean 61) groups were balanced in educational background (young: 55% high-school; 45% university; older: 50% high-school, 50% university). Both groups were tested on a Test Of English as Foreign Language (TOEFL) as part of a standard test of general knowledge. The Quick test format used in the study was composed of 10 words, each followed by a four-alternative multiple choice question probing the word’s meaning. The vocabulary test was administered after the free recall memory task. The mean score for young adults was 6.05 (SD = 2.19) and for the older adults it was 5.4 (SD = 2.52). No group differences were present (*t* < 1). Thus, any group differences in memory recall or clustering cannot be attributed to group differences in background vocabulary. The study was approved by the Birkbeck ethics committee.

### 2.2. Materials

For the memory task, we used a pool of 240 words consisting of common one- and two-syllable nouns with 90 pairs of weak associates (e.g., “stomach–liver”, see [28]); weak associates were used in order to avoid guessing strategies. The words have a mean written word frequency of 66.9 per million, and the average associative strength was 0.15 (SD = 0.14; [31]). Each memory list consisted of 12 words and was constructed in accordance with one of four separation conditions. The five lists in the unrelated condition contained twelve words that did not have a semantic relation. We created three types of related lists by varying the number of intervening unrelated n items separating the members of a pair. We used separation-0, -1, and -5. With separation-0, the members of an associated pair, a1–a2, appeared in temporally adjacent list positions (e.g., a1, a2, b1, b2, c1, c2, d1, d2, e1, e2, f1, f2). In separation-1, the pair members were separated by one unrelated word, which was a member of a different pair (e.g., a1, b1, a2, b2, c1, d1, c2, d2, e1, f1, e2, f2), whereas in the separation-5 condition, five words separated the pair members (e.g., a1, b1, c1, d1, e1, f1, a2, b2, c2, d2, e2, f2). There were a total of 23 lists: three practice trials and 20 experimental trials.

### 2.3. Procedure

Participants were tested individually in noise-attenuated conditions and presented with the computerised memory task followed by the vocabulary test. Instructions were presented on the computer screen as well as verbally to ensure comprehension. Presentation of the memory test was visual. Participants were seated in front of the monitor, fixating the centre of the screen. Each trial started with a row of three question marks in the middle of the screen. These were replaced by words presented at a rate of one word per second. Participants were required to read each word silently and immediately after the final word to perform the arithmetic distractor task. This distractor task consisted of a mix of 12 additions and subtractions of the form A +/− B = C, where A, B and C were positive single digit numbers such as e.g., “1 + 2 = 3”. The participant was instructed to quickly press the “k” (when correct) or “s” button on the keyboard to indicate the accuracy of the mathematical expression. A row of three question marks prompted the participants to recall aloud as many words from the list as possible, in any order. The experimenter wrote down the recalled items.

### 2.4. Semantic Clustering Scores

Semantic clustering was estimated using the pair frequency score, which is identical to the original clustering metric proposed by Bousfield and Bousfield [32]. This metric looks at the memory recall protocol and focuses on the number of within-category repetitions. To illustrate, consider a two-category word list, made of *cat, dog, rabbit, fork, knife, spoon*, with a recall output of *cat, dog, fork, knife, rabbit*. The pair frequency is the difference between the observed number of within-cluster transitions, which is 2 (cat → dog, fork → knife) and the expected number of within-cluster transitions, which is calculated using the following formula:

(1) $$\overset{N_{c}}{\underset{i=1}{\sum }}\frac{n_{i}\left(n_{i}-1\right)}{r}$$

where *n_i_* is the number of correct words for category *i*, out of *N_c_* recalled categories, and *r* is the total recall. In the example, *i* = 2, *n*_1_ = 3 (cat, dog, rabbit), *n*_2_ = 2 (fork, knife), and *r* is 5. This leads to an expected number of within-cluster transitions of 3 × 2/5 + 2 × 1/5 = 1.6, and the overall clustering score of 2 − 1.6 = 0.4.

In addition, we also estimated semantic clustering, using the California Verbal Learning Test (CVLT: [33]) measure. As the results are the same, we only report the pair-frequency measure here. 

## 3. Results

**Figure 1:**
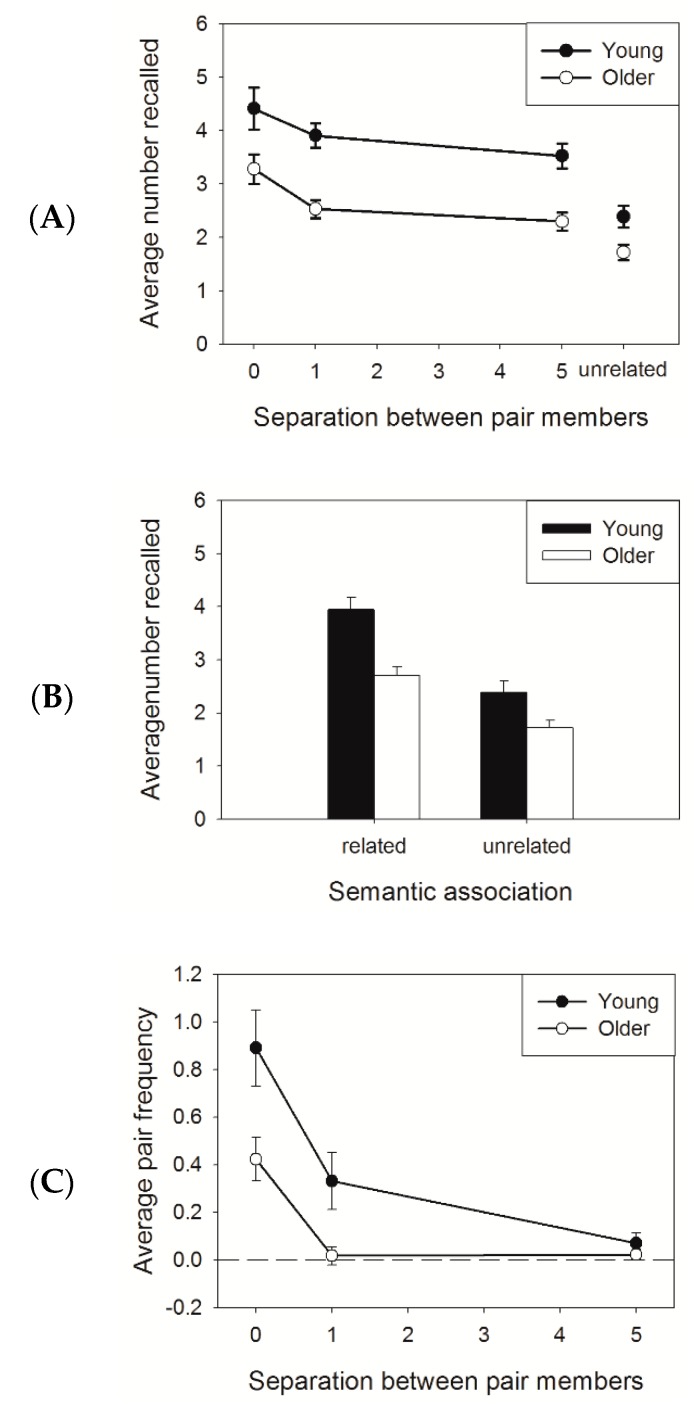
(**A**) Average number of words recalled as a function of the separation between the members of an associated pair and age. The separations in the related condition are presented. (**B**) The same as in panel A, but the related condition is averaged across separations. (**C**) Clustering scores in the related conditions as a function of the separation between pair members and age. The error bars represent standard error of the mean.

Figure 1A,B show the average number of words recalled in the four conditions (three separations and one unrelated) and for the related (averaged across separation) and unrelated conditions, for young and older adults, respectively. Participants recalled more words in the related than in the unrelated condition. Younger adults reported more words than older adults, especially in the related condition. A 2 (age: young, older) × 2 (association: related, unrelated) mixed ANOVA revealed a main effect of age (F (1,38) = 15.83, MSe = 1.16, *p* < 0.001, *η*^2^ = 0.29), a main effect of association (F (1,38) = 102.85, MSe = 0.31, *p* < 0.001, *η*^2^ = 0.73), and an interaction (F (1,38) = 5.23, MSe = 0.31, *p* < 0.05, *η*^2^ = 0.12). The interaction was due to a larger increase in total recall with related lists for younger than for older adults (*t* (38) = 2.29, *p* < 0.05). The same conclusions were obtained when the ANOVA was limited to unrelated versus separation-5.

Both groups showed equivalent decrease in recall performance with increasing separation between pair members. A 2 (age: young, older) × 3 (separation: 0, 1, 5) mixed ANOVA revealed a significant main effect of age (F (1,38) = 18.42, MSe = 2.52, *p* < 0.001, *η*^2^ = 0.33) and a main effect of separation (F (2,76) = 12.25, MSe = 0.74, *p* < 0.001, *η*^2^ = 0.24). The interaction between age and separation was not significant (F < 1).

We computed, for each separation, a semantic clustering score. Several metrics have been developed to measure semantic clustering [20,34]. Here, we use the pair frequency, which is identical to the original clustering metric proposed by Bousfield and Bousfield [32]. If, for example, a participant saw the words light_1_ man_2_ candle_1_ father_2_ hat_3_ river_4_ cap_3_ lake_4_ door_5_ thief_6_ wall_5_ crook_6_ (separation-1) and then reported lake_4_ river_4_ light_1_ man_2_ candle_1_ (numbers are only added for illustration and were not present in the experiment), the clustering score would be 0.20 (OBS − EXP = 1 − (2 × 1/5 + 2 × 1/5 + 1 × 0/5) = 0.2). See Methods for details.

Figure 1C presents the clustering scores for each separation. Younger adults (filled circles) clustered the members of a pair more often than older adults (open circles). In addition, increasing the separation between pair members during encoding lowered the clustering effect for both age groups. A 2 (age: younger, older) × 3 (separation: 0, 1, 5) mixed ANOVA revealed a significant main effect of separation (F (2,76) = 39.39, MSe = 0.11, *p* < 0.001, *η*^2^ = 0.51) and age (F (2,76) = 7.34, MSe = 0.31, *p* < 0.01, *η*^2^ = 0.16) and an interaction between separation and age (F (2,76) = 4.29, MSe = 0.11, *p* < 0.05, *η*^2^ = 0.10). This interaction was due to an absence of an age effect for separation 5 (*p* > 0.34). Also, older adults did not cluster the words when the pair members were separated by one or more unrelated words (all *p*s > 0.10).

## 4. Discussion

We tested for age-related differences in memory recall of word lists made of associated pairs, with various degrees of separation (number of unrelated intervening words) between the pair members. First, we found that lists containing associated pairs are recalled better than lists of un-associated words in both age groups and that this difference is larger for young adults. In particular, we found only small age differences for lists of unrelated words (0.67 words) and we found larger age differences for lists containing associated pairs (1.32 words). This interaction is also present when comparing the unrelated and separation-5 conditions. As word associates, which are separated by five intervening unrelated words, are not likely to co-occupy working memory, this interaction is likely to indicate a retrieval deficit: younger participants might be better in using the last word recalled, as a cue to memory search [2].

Second, we found that for both age groups, the association effect (better recall of associated than unrelated words) also depends on the separation of the pair members in the memory list, with better performance at short separation. This replicates the results by Glanzer [26], indicating that the encoding of the list is more effective when associated pairs co-occupy a capacity-limited working memory [27,29]. If older participants have reduced working memory capacity, one could expect that they will benefit less of associated pairs at separation-1; in both conditions, the pairs are unlikely to co-occupy working memory. Although the difference in total recall for separations 1 and 5 was numerically larger for young than for older participants, this difference was not statistically significant.

Third, we examined the clustering measures, of the two age groups, at each level of separation. Participants produced more clustered output, at short separations, consistent with an encoding mechanism (co-occupation of the associates in working memory, enhances their association). As we predicted, younger participants produced more clusters at separation-1 than at separation-5, consistent with an age-dependent working memory-assisted encoding. The clustering frequency for the old group at separation-1 was not different from that at separation-5, which was at chance level. Thus, older adults show reduced beneficial effect of closer temporal proximity, as expected from a reduced encoding of relations between words that co-occupy working memory. The absence of clustering at separation-1 critically supports the suggestion that older adults’ working memory capacity is just too small to have both members of a pair active simultaneously after a single intervening unrelated item. This prevents the detection of the semantic similarity and the associative boost that is needed for these items to be recalled consecutively. While we report the pair frequency measure for semantic clustering, we also looked at other clustering measures such as the one used for the CVLT. Age-effects at separation-1 were present in these measures at separation-1, with older adults showing no clustering beyond what is expected by chance.

We suggest that the results indicate age effects in both the retrieval and encoding of a list of words, which are due to a reduced ability to encode relations between list-words (see also [35] for similar findings using a memory recognition paradigm). This is consistent with previous age studies of clustering, which used the CVLT [33], reporting marked age-related decline [25]. In a previous study, we have shown that ageing is associated with a significant deficit in a test that shares some features with the CVLT—The conceptual-span task [36]. Furthermore, our results parallel those obtained in memory studies in frontal patients, who do not show enhanced recall with related, compared with unrelated word lists [37], and who do not spontaneously utilise cues to enhance their memory encoding and retrieval, and show reduced semantic clustering [12]. These frontal memory deficits are well accounted for by neuropsychological models of memory, such as Hemispheric Encoding-Retrieval Asymmetry (HERA) [38], which postulate a role for the left PFC in semantic processing and memory encoding and by computational models that unpack the PFC mechanisms involved in memory encoding and retrieval [24,39]. For example, in the Categorization–Activation–Novelty (CAN; [24]) model, the PFC detects the semantic differences and similarities among list items that co-occupy WM. Semantic associates get an encoding boost, but only if their relatedness has been detected. 

We believe that these similarities are explained best by the hypothesis that ageing deficits in memory are caused by a frontal mechanism [7,40]. As previously reviewed, this hypothesis is supported by neuropsychological, neurological, and neuroimaging evidence, showing changes in frontal lobe structures and functioning with advancing age. This interpretation is also consistent with the finding that older adults are less likely to form rich, elaborative memory traces [5]. The results presented here provide convergent evidence within a single paradigm suggesting that age-related declines in total recall are associated with a decrease in the use of associative information during both encoding and retrieval.
